# Lipomatous Variant of Solitary Fibrous Tumor With CD34 Negativity: A Case Report

**DOI:** 10.7759/cureus.106134

**Published:** 2026-03-30

**Authors:** Mohammed Bakhti, Anass Haloui, Nassira Karich, Amal Bennani

**Affiliations:** 1 Department of Anatomopathology, Faculty of Medicine and Pharmacy Oujda, Oujda, MAR

**Keywords:** lipomatous, rare, solitary fibrous tumor, the soft tissues, tumoral

## Abstract

The solitary fibrous tumor (SFT) in its lipomatous variant is very rare, with only a few cases described in the literature. It is a myofibroblastic mesenchymal tumor with intermediate behavior. We report the case of a 33-year-old patient with no notable pathological history, admitted following the incidental discovery of a dorsal mass involving the soft tissues, whose radiological examination suggested a schwannoma. The patient underwent a surgical biopsy showing a tumoral proliferation composed of spindle cells within mature adipose tissue. Immunohistochemical studies were necessary to rule out differential diagnoses before establishing the final diagnosis.

## Introduction

The solitary fibrous tumor (SFT) is a generally rare fibroblastic tumor proliferation; only 51 cases have been described in the literature. Initially identified in the pleura [1,2,3], extrapleural locations are now more predominant, with 10% to 15% of cases occurring in the trunk region. Clinically, it presents as an asymptomatic mass, with or without mass effect. While this tumor is largely benign, it can lead to distant metastases or locally recurrent forms in 12% of cases [4]. Histologically, it is a well-circumscribed mass composed of spindle cells associated with hemangiopericytoma-like vascularization. Several histological variants exist, posing a challenge for differential diagnosis with other fibroblastic tumors, particularly in our case, which corresponds to the lipomatous variant of SFT. This highlights the importance of immunohistochemical study, primarily based on the anti-STAT6 antibody, which is the most specific marker.

## Case presentation

**Figure 1:**
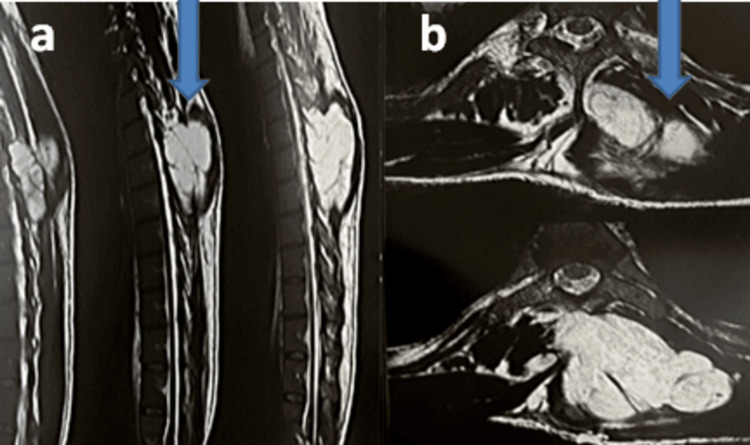
Injected dorsal MRI in sagittal (a) and axial (b) sections Dorsal tumoral process, developing intradurally extramedullarily from D6 to D8, extending to the adjacent left foramina with corresponding costovertebral osteolysis, and significant extension to the paravertebral soft tissues from D4 to D8, with frank T2 hypersignal, first suggesting a schwannoma.

We report the case of a 33-year-old man with no significant personal or family history and no evidence of altered general condition, who presented with an indolent dorsal mass discovered incidentally during a bloodletting session. This prompted the patient to undergo a dorsal MRI, which revealed a large soft-tissue mass from D6 to D8, with intracanalar invasion (Figure 1). He was subsequently referred to the neurosurgery department for management, where he underwent a surgical biopsy complicated by sudden hemorrhagic shock requiring immediate resuscitation. The specimen was then sent to our pathology department for histological confirmation.

**Figure 2:**
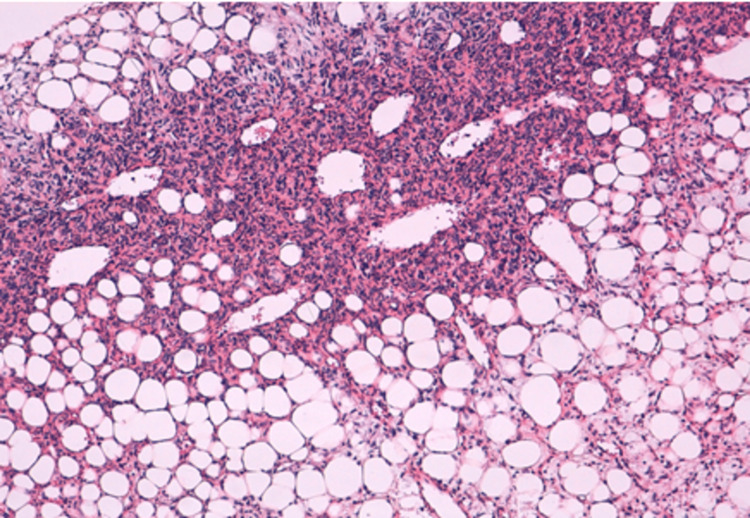
Tumor proliferation consisting of a double component (low magnification).

**Figure 3:**
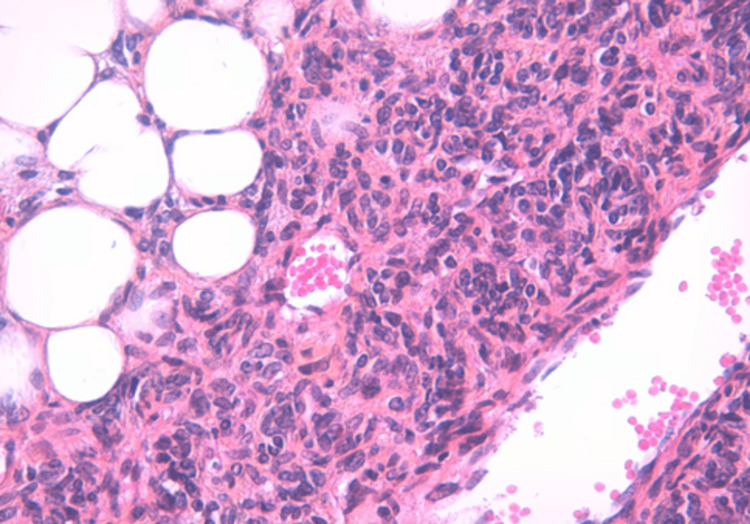
Tumor proliferation made up of monotonous, slightly atypical spindle cells associated with regular adipocytes (high magnification).

Histological examination revealed a highly cellular, tumoral proliferation organized in interlacing fascicles, composed of slightly atypical spindle cells with ovoid nuclei finely nucleolated, the cytoplasm abundant and eosinophilic. It was associated with the presence of regular adipose tissue enclaved within the tumoral proliferation. Furthermore, there were a few thin-walled vessels of hemangiopericytoma type, arranged within the tumoral proliferation (Figures 2, 3).

**Figure 4:**
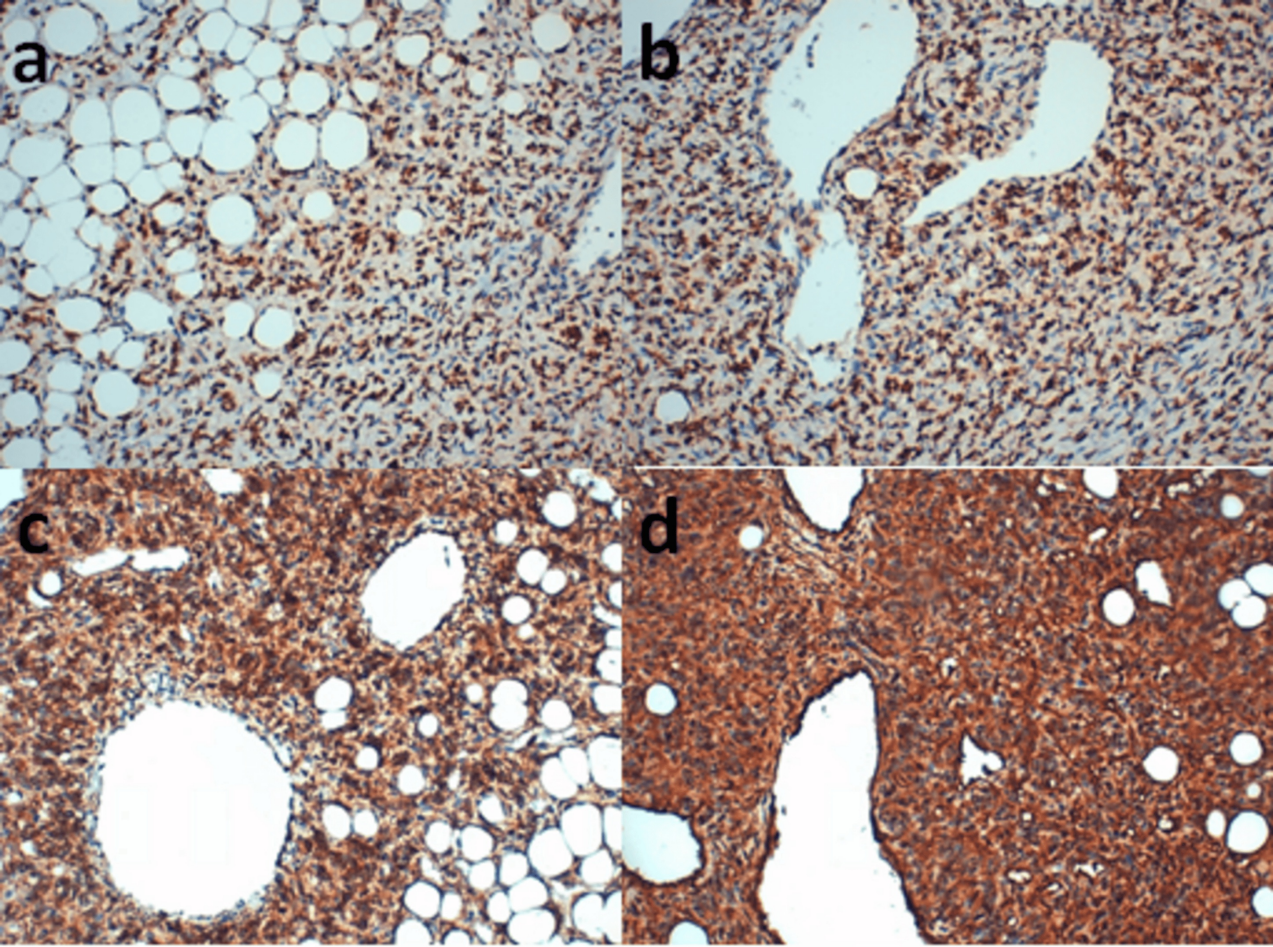
Immunohistochemical study showing positive staining of the tumor proliferation by various antibodies. a, b: STAT6+; c: BCL2+; d: CD99+ at high magnification

**Figure 5:**
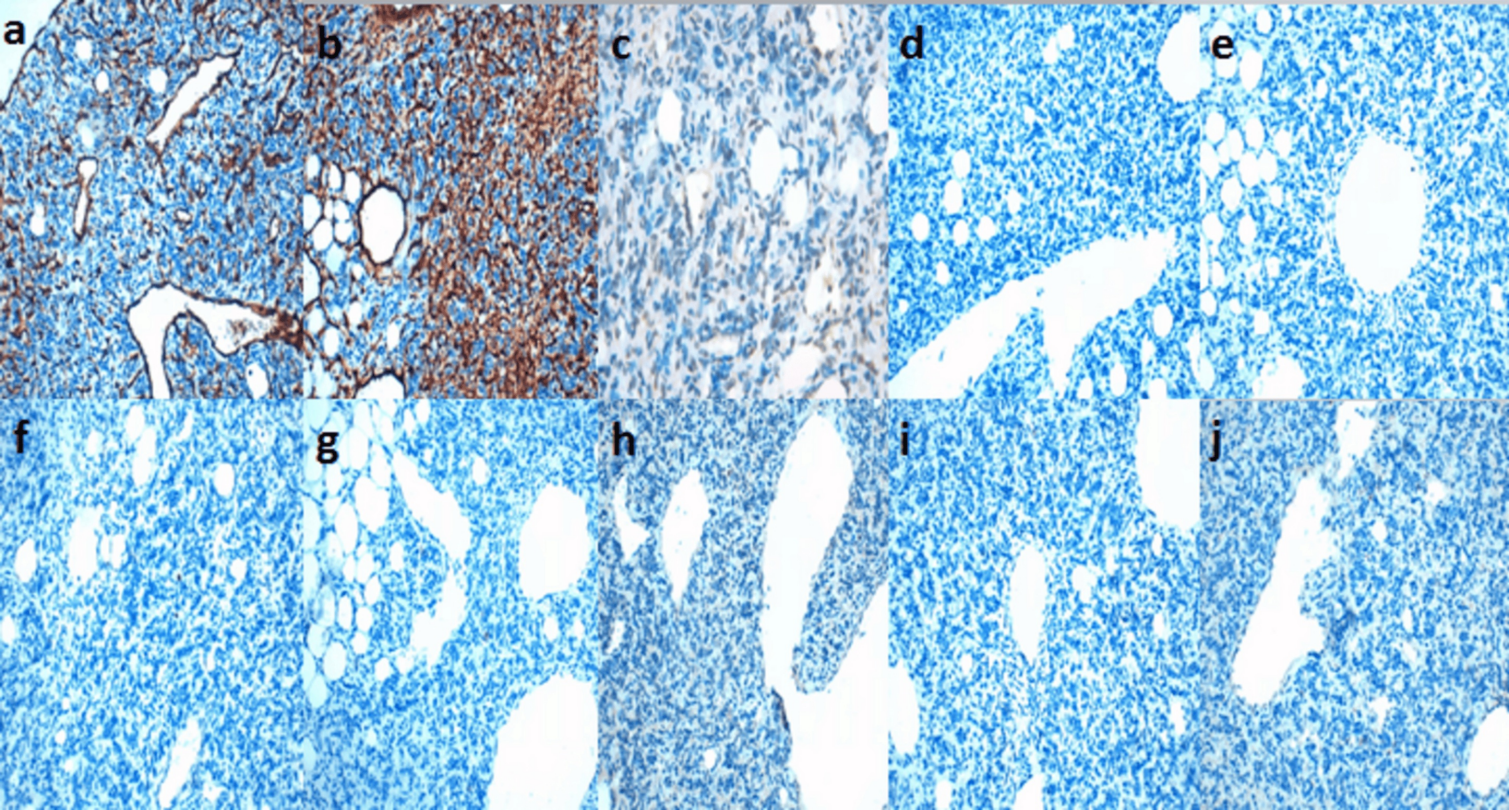
Immunohistochemical study showing an absence of staining of the tumor proliferation by various antibodies. a: CD31-; b: CD34-; c: MDM2-; d: CDK4-; e: Myogenin-; f: S100-; g: CK-; h: Desmin-, i: EMA-; j: GFAP- at high magnification

The results of the immunohistochemical study (Figures 4, 5) further clarified the tumor's profile. The tumor cells exhibited diffuse positive staining for STAT6, Bcl2, and CD99. Conversely, the cells were negative for CD34 and CD31, which highlighted the internal vasculature rather than the tumor itself. Additionally, the study confirmed an absence of staining for a broad panel of markers, including MDM2, CDK4, Myogenin, S100, Pancytokeratin, Desmin, EMA, and GFAP, effectively ruling out several alternative diagnoses.

In view of these concordant histological and immunohistochemical data, the diagnosis of solitary fibrous tumor in its lipomatous variant was retained.

## Discussion

The solitary fibrous tumor (SFT) belongs to the category of fibroblastic mesenchymal tumors. It was first described in the pleura [5, 6]. It is characterized by the fusion of the *NAB2-STAT6 *gene, located on the chromosomal region 12q13 [7]. It occurs at any age, but the most affected age group is between 40 and 70 years. Clinically, it presents as a well-defined, painless mass with slow progression, which most often develops in soft tissues, but there are other fairly frequent extrapleural locations, such as the meninges, abdomen, head, and neck [6].

Histologically, it is a highly cellular tumoral proliferation composed of fibroblastic cells, associated with hemangiopericytoma-like vascularization, without tumoral necrosis or marked pleomorphism, and with a low mitotic index [8]. Immunohistochemical study was positive for anti-CD34 antibody (71.4%), anti-Bcl2 (77.1%), anti-CD99 (60%), and anti-STAT6 (88.6 to 91%), the latter being the most specific, which confirms the diagnosis [4,9, 10]. A study by Barthelmeß et al. showed an estimated positivity rate of 100% by an immunohistochemical study for STAT6, whereas detection of the gene fusion is only found in 92% of cases by RT-PCR [11].

The lipomatous variant of solitary fibrous tumor (LSFT) is generally rare. It features regular and mature adipose tissue, with the same clinical, morphological, immunohistochemical, and cytogenetic characteristics as the classic form [2]. This variant poses differential diagnostic challenges, particularly with well-differentiated and dedifferentiated liposarcoma, which are characterized by positive staining for anti-CDK4 and anti-MDM2 antibodies, cytoplasmic and nuclear staining for the anti-STAT6 antibody, and absence of staining for the anti-CD99 antibody. In contrast, in our variant, there is an absence of staining for anti-CDK4 and anti-MDM2 antibodies, positive nuclear staining for the anti-STAT6 antibody, and diffuse positive staining for the anti-CD99 antibody [12-17].

The prognosis is excellent for forms lacking criteria of malignancy, which are necrosis, atypia, frequent mitoses, and infiltration [2]. The metastatic risk is assessed by Demicco et al., based on four criteria: age ≥ 55 years, mitoses (≥ 2/mm²), necrosis, and tumor size. According to this grading, the tumor can be classified as low risk, intermediate, or high [18].

Treatment relies on wide excision of the tumor; adjuvant chemotherapy and radiotherapy have no role in the classical forms [19].

## Conclusions

According to the World Health Organization, a solitary fibrous tumor is considered a fibroblastic tumor with intermediate behavior. The lipomatous variant poses a differential diagnosis challenge, but its prognosis remains similar to the classical form. Surveillance is recommended to monitor for recurrence, especially for forms with malignant characteristics.
